# Targeted mutagenesis and base editing using engineered *Brevibacillus laterosporus* Cas9 with expanded target scope in rice

**DOI:** 10.5511/plantbiotechnology.26.0218a

**Published:** 2026-06-25

**Authors:** Katsuya Negishi, Hiroshi Nishimasu, Osamu Nureki, Seiichi Toki, Masaki Endo

**Affiliations:** 1 Institute of Fruit Tree and Tea Science, National Agriculture and Food Research Organization, 2-1 Fujimoto, Tsukuba, Ibaraki 305-8605, Japan; 2 Structural Biology Division, Research Center for Advanced Science and Technology, The University of Tokyo, 4-6-1 Komaba, Meguro-ku, Tokyo 153-8904, Japan; 3 Department of Chemistry and Biotechnology, Graduate School of Engineering, The University of Tokyo, 7-3-1 Hongo, Bunkyo-ku, Tokyo 113-8656, Japan; 4 Inamori Research Institute for Science, 620 Suiginya-cho, Shimogyo-ku, Kyoto, Kyoto 600-8411, Japan; 5 Department of Biological Sciences, Graduate School of Science, The University of Tokyo, 7-3-1 Hongo, Bunkyo-ku, Tokyo 113-0033, Japan; 6 Department of Life Science, Faculty of Agriculture, Ryukoku University, 1-5 Yokotani, Seta, Oe-cho, Otsu, Shiga 520-2194, Japan; 7 Graduate School of Nanobioscience, Yokohama City University, 22-2, Seto, Yokohama, Kanagawa 236-0027, Japan; 8 Kihara Institute for Biological Research, Yokohama City University, 641-12 Maioka-cho, Yokohama, Kanagawa 244-0813, Japan; 9 Institute of Agrobiological Sciences, National Agriculture and Food Research Organization, 3-1-3 Kannnondai, Tsukuba, Ibaraki 305-8604, Japan

**Keywords:** CRISPR/Cas9, enBlCas9, genome editing, *Oryza sativa*, proxy-CRISPR

## Abstract

The CRISPR/Cas9 system is now widely used for precise genome editing in many crop species, allowing targeted mutagenesis and precise base substitution. While CRISPR/Cas9-mediated genome editing is highly accurate, a limitation of this technology is the requirement for an appropriate protospacer adjacent motif (PAM) for target site recognition. Cas9 from *Brevibacillus laterosporus* (BlCas9) has been reported to recognize N_4_CNDD PAMs with a pronounced preference for A at the 7th and 8th positions of the PAM, and is used for genome editing in maize and human cells. Recently, the enhanced BlCas9 (enBlCas9) variant was reported that includes two amino acid mutations in the PAM-interacting domain of BlCas9 and exhibits enhanced genome editing activity with an expanded target scope in vitro and in human cells. Here, we demonstrate the BlCas9- and enBlCas9-mediated genome editing in rice. Both BlCas9 and enBlCas9 can introduce targeted mutations in rice, and enBlCas9 can broaden the target scope with a non-A residue at the 7th and 8th bases of the PAM. Furthermore, enBlCas9 is applicable for precise base editing with extended target sites, such as C-to-T and A-to-G base conversions. In addition, we developed and validated a proximal CRISPR targeting method (proxy-CRISPR) in which nuclease-dead SpCas9 (SpdCas9) bound near the target sites can improve the genome editing activity of BlCas9 and enBlCas9. These enBlCas9-based genome editing technologies are expected to broaden the scope of efficient targeted mutation and precise base editing in rice.

## Introduction

Clustered regularly interspaced short palindromic repeat/CRISPR-associated protein 9 (CRISPR/Cas9) technology is now widely used as a tool for genome editing in various plant species, including many crops. The Cas9 and single guide RNA (sgRNA) complex binds at the target DNA at a sequence complementary to the sgRNA and introduces targeted mutations through producing a DNA double-stranded break (Jinek et al. 2012). Cas9 requires the presence of a specific DNA sequence called a protospacer adjacent motif (PAM) for DNA recognition. For example, the most widely used *Streptococcus pyogenes* Cas9 (SpCas9) recognizes the PAM sequence NGG (N means any nucleotide). This PAM requirement limits the targetable range of CRISPR/Cas9 genome editing. Recent technological advancements have allowed more precise genome editing such as base substitutions, without the need for DNA double-stranded break at the target site. The fusion of Cas9 nickase (nCas9) or catalytically dead Cas9 (dCas9) protein with the DNA deamination function of cytidine deaminase or adenosine deaminase generates cytosine base editors (CBEs) or adenine base editors (ABEs), which can introduce C to T or A to G substitutions, respectively (Gaudelli et al. 2017; Nishida et al. 2016). The base editing windows of CBEs and ABEs are determined by both the type of deaminase and the distance from the PAM (Huang et al. 2021). Therefore, genome editing technologies that are less restricted by specific PAM sequences are important to allow precise genome modification in plants. Since each Cas ortholog recognizes a specific sequence as a PAM, developing a genome editing toolbox using various Cas orthologs can broaden the range of targetable genomic loci. Several Cas9 orthologs has been used for plant genome editing such as *Staphylococcus aureus* Cas9 (SaCas9) (Kaya et al. 2016) and *Streptococcus thermophiles* Cas9 (StCas9) (Steinert et al. 2015). In addition to Cas9 orthologs, the utilization of an alternative RNA-guided endonuclease has also been reported for rice genome editing such as *Francisella novicida* Cas12a (FnCas12a) (Endo et al. 2016) and SpCas12f (Sukegawa et al. 2023). The development of Cas9 variants is also an effective approach to enhance the flexibility of genome editing. Several engineered SpCas9 variants with altered PAM recognition have been developed using various strategies, such as directed evolution (Hu et al. 2018; Miller et al. 2020) and rational engineering by crystal structure analysis (Nishimasu et al. 2018; Walton et al. 2020). These engineered Cas9 variants have been used for precise genome editing in plants (Endo et al. 2019; Negishi et al. 2019; Ren et al. 2021).

Cas9 from *Brevibacillus laterosporus* SSP360D4 (BlCas9, also known as BlatCas9) was reported as a Cas9 ortholog that recognizes N_4_CNDD (D is A, G or T) as a PAM sequence and induces targeted mutations in maize and human cells (Gao et al. 2020; Karvelis et al. 2015). Since most Cas9 orthologs recognize G-rich sequences as the PAM, the development of genome editing technology using BlCas9 could alleviate PAM limitations. However, these experiments showed that the genome editing activity of BlCas9 is high at N_4_CNAA PAM target sites but weak at other N_4_CNDD PAM targets. The mutation efficiency of BlCas9 depends on the target locus and is sometimes low compared with SpCas9 (Gao et al. 2020; Karvelis et al. 2015). Recently, Nakane et al. (2024) reported the crystal structure of the BlCas9-sgRNA-target DNA complex and confirmed that BlCas9 recognizes N_4_CNDN PAM with a pronounced preference for A at positions 7 and 8. They also developed an enhanced BlCas9 (enBlCas9) variant based on the structure. The enBlCas9 includes two amino acid substitutions (E904R and T1025A) of BlCas9 and exhibits enhanced activity and relaxed PAM preference at the 7th and 8th positions.

The use of Cas9 orthologs and engineered Cas9 variants can extend the targetable range of genome editing; however, some of these variants show low genome editing activity in vivo, even at the preferred target sites (Endo et al. 2019; Nakane et al. 2024). Mutation efficiency by Cas9 enzymes is affected by in vivo DNA status, including chromatin structure (Horlbeck et al. 2016). The proximal CRISPR targeting method (proxy-CRISPR) has been developed as a way to reduce the inhibitory effect of chromatin structure on genome editing (Chen et al. 2017). proxy-CRISPR uses an additional dCas9 that binds at proximal locations of the target site and can enhance the Cas9 activity by altering the local accessibility of Cas9 in vivo (Chen et al. 2017). Modulating chromatin accessibility using proximal dCas9 to increase genome editing efficiency has also been reported in rice (Liu et al. 2019).

In the present study, we established BlCas9- and enBlCas9-mediated genome editing in rice and showed that enBlCas9 can introduce targeted mutation to more expanded target sites than BlCas9. We also found that enBlCas9-based CBEs and ABEs is applicable for base editing with relaxed PAM. In addition, the combination of proxy-CRISPR and enBlCas9 could provide more efficient genome editing in rice. Overall, enBlCas9-based genome editing tools will support both plant breeding and basic research.

## Materials and methods

### Vector constructions

#### sgRNA expression cassette in cloning vectors

The SpCas9 sgRNA cloning vector (pUC_OsU6:SpCas9-sgRNA) was used in a previous study (Mikami et al. 2015). The scaffold sequence for BlCas9 sgRNA defined in previous reports (Karvelis et al. 2015) was synthesized by GeneArt (Thermo Fisher Scientific, Waltham, MA, USA) and replaced the SpCas9-sgRNA scaffold sequence in construct pUC_OsU6:BlCas9-sgRNA. These vectors were linearized using restriction enzyme BbsI (New England Biolabs, Ipswich, MA, USA) and the 20-nt (SpCas9 target sites) or 22-nt (BlCas9 target sites) annealed oligonucleotides were ligated between the *Oryza sativa* U6-2 small nuclear RNA promoter (pOsU6) and the sgRNA scaffold sequence of these vectors. The sgRNA sequence for BlCas9 was designed with a 22-nt protospacer sequence, the optimal length for BlCas9 (Nakane et al. 2024), and a 147-nt scaffold sequence containing two substitutions (T to G at position 100 and T to C at position 158) to remove a potential U6 RNA polymerase III termination sequence (Karvelis et al. 2015).

#### BlCas9, enBlCas9, and SpRY expression binary vectors for target mutagenesis

**Figure 1:**
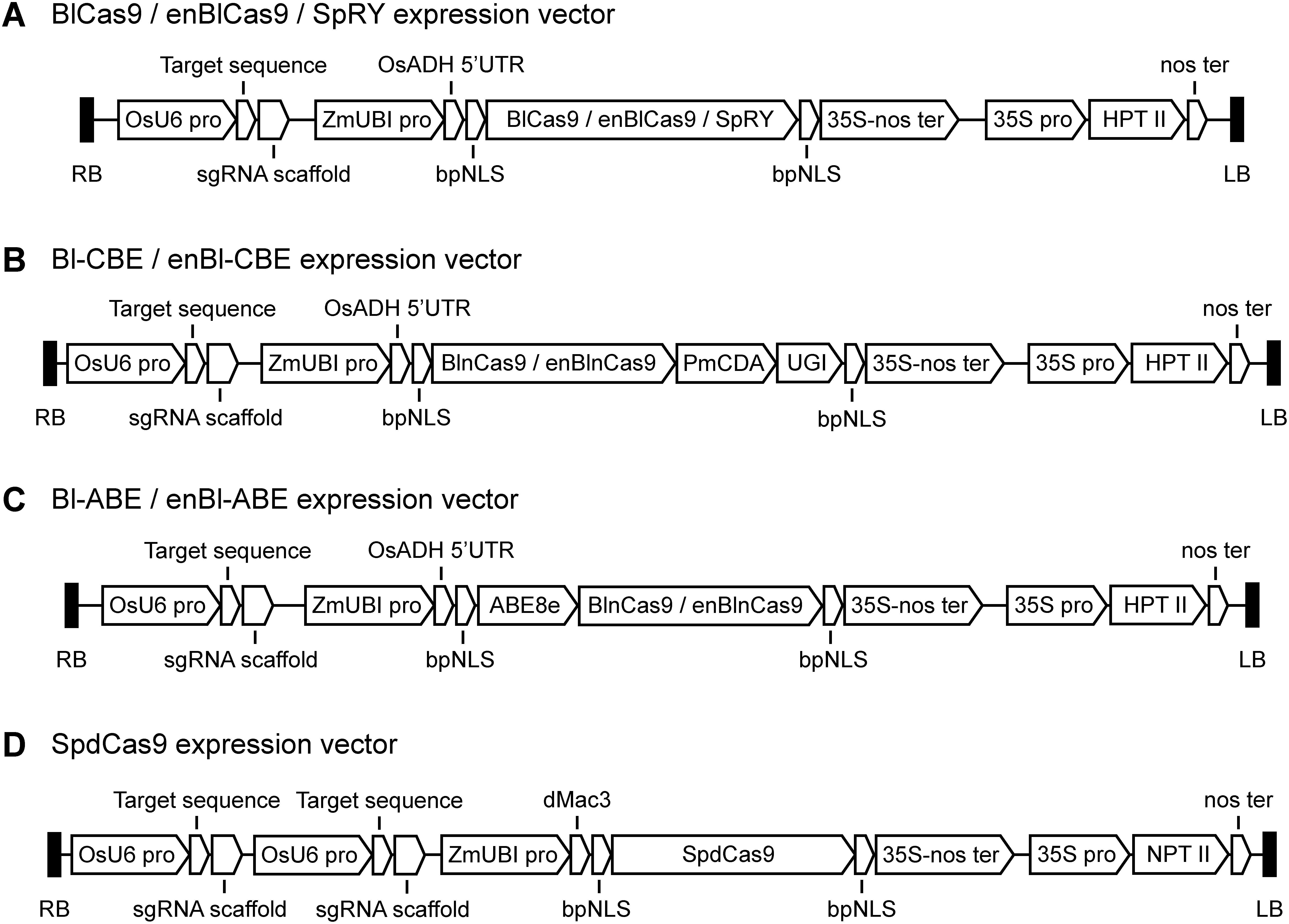
Figure 1. Vector constructs using in this study. (A) BlCas9/enBlCas9/SpRY expression vectors for targeted mutagenesis. (B) Bl-CBE/enBl-CBE expression vectors for cytosine base editing. (C) Bl-ABE/enBl-ABE expression vectors for adenine base editing. (D) SpdCas9 expression vector for proxy-CRISPR. These vectors were constructed in the pPZP vector backbone. The figures show the transfer DNA region between the right border (RB) and the left border (LB). OsU6 pro, *O. sativa U6-2* small nuclear RNA promoter; sgRNA scaffold, sgRNA scaffold sequence of SpCas9 or BlCas9; ZmUBI pro, *Zea mays* *polyubiquitin 1* promoter; OsADH 5′ UTR, *O. sativa ALCOHOL DEHYDROGENASE* 5 prime untranslated region; bpNLS, bipartite nuclear localization signal; BlCas9/enBlCas9/SpRY, BlCas9 or enBlCas9 or SpRY coding sequence; 35S-nos, double terminator of *cauliflower mosaic virus 35S* terminator and *Agrobacterium tumefaciens* *nopaline synthase* terminator; 35S pro, c*auliflower mosaic virus 35S* promoter; HPT II, *hygromycin B phosphotransferase II* coding sequence; BlnCas9/enBlnCas9, BlnCas9 or enBlnCas9 coding sequences which were introduced D8A mutation; PmCDA, *Petromyzon marinus cytidine deaminase 1* coding sequence; UGI, uracil DNA glycosylase inhibitor coding sequence; ABE8e, *O. sativa* codon-optimized engineered adenosine deaminase domain; dMac3, *O.sativa Mac3* 5 prime untranslated region; SpdCas9, SpdCas9 coding sequence which was introduced D10A and N863A mutation; NPT II, *aminoglycoside phosphotransferase II* coding sequence.

The transfer DNA region of BlCas9, enBlCas9, and SpRY expression binary vectors consists of three parts: a sgRNA expression cassette, a Cas9 expression cassette, and a hygromycin resistance selective marker gene (*hygromycin B phosphotransferase II*; *HPT II*) expression cassette. *O. sativa* codon-optimized BlCas9 and SpRY sequences with a bipartite nuclear localization signal (bpNLS) (Koblan et al. 2018) were synthesized by GeneArt, and E904R and T1025A mutations were introduced into the BlCas9 fragment to create the enBlCas9 sequence (Nakane et al. 2024). To regulate the expression of Cas9, *Zea mays polyubiquitin 1* promoter (pZmUBI), *O. sativa ALCOHOL DEHYDROGENASE* 5′ UTR region (OsADH 5′ UTR), and double terminator of cauliflower mosaic virus 35S (35S) terminator and *Agrobacterium tumefaciens* nopaline synthase (nos) terminator (35S-nos ter) were used. These regulatory elements of Cas9 and the selection marker cassette were PCR-amplified from pZH_OsU6sgRNA_SpCas9-WT (Endo et al. 2019) using KOD One PCR Master Mix (TOYOBO, Osaka, Japan). These DNA fragments were cloned into a pPZP200 vector using an In-Fusion HD Cloning Kit (Takara Bio, Shiga, Japan). Subsequently, a sgRNA expression cassette was inserted into the binary vector using restriction enzymes AscI and PacI (New England Biolabs) to generate BlCas9, enBlCas9, and SpRY expression vectors (Figure 1A).

#### CBEs and ABEs using BlCas9 and enBlCas9 expression vectors for base editing

We introduced a D8A mutation in the BlCas9 and enBlCas9 to generate BlnCas9 and enBlnCas9 respectively. To develop the CBE, we introduced *Petromyzon marinus* cytidine deaminase 1 (PmCDA1) and uracil DNA glycosylase inhibitor (UGI) sequences (Endo et al. 2019; Shimatani et al. 2017) between the BlnCas9/enBlnCas9 and bpNLS at the C-terminal region. To create an ABE using BlnCas9 and enBlnCas9, *O. sativa* codon-optimized engineered adenosine deaminase domain ABE8e (Richter et al. 2020), containing ABE8e and a 32aa linker sequence, was synthesized by GeneArt. The ABE8e fragment was inserted between the 5′ UTR of OsADH and BlnCas9 or enBlnCas9 using an In-Fusion HD Cloning Kit. Subsequently, a sgRNA expression cassette was inserted into the binary vector using restriction enzymes AscI and PacI to generate CBEs and ABEs using BlCas9 and enBlCas9 expression vectors (Figure 1B, C).

#### SpdCas9 expression vector for proxy-CRISPR

To create a SpdCas9 expression vector (Figure 1D), we used *O. sativa* codon-optimized SpCas9 sequence (Mikami et al. 2015) as a template and introduced D10A and N863A mutations (Nishimasu et al. 2014) by PCR using KOD One PCR Master Mix. The SpdCas9 fragment was replaced with the BlCas9 sequence in the BlCas9 expression vector. To improve SpdCas9 protein translation level, we used translational enhancer OsMac3 mRNA 5′ UTR region (dMac3) instead of the OsADH 5′ UTR (Kusano et al. 2018). The *HPT II* coding sequence was then pulled out and replaced with the geneticin reagent (G-418) resistance selective marker gene (*neomycin phosphotransferase II*; *NPT II*) coding sequence, which is a type of aminoglycoside phosphotransferase that provides a G-418 resistant phenotype. The tandemly SpCas9-sgRNA expression cassettes were introduced into the binary vector according to a previously described method (Mikami et al. 2016).

### Plant material and rice transformation

*O. sativa* cv. Nipponbare line was used as wild type rice in this study. The *Agrobacterium tumefaciens*-mediated transformation of rice using scutellum-derived calli was performed as described previously (Endo et al. 2019). Transformed calli were grown on a callus-induction medium containing 50 mg l^−1^ hygromycin B (FUJIFILM Wako Pure Chemical, Osaka, Japan) for DNA cleavage and base editing experiments, or 50 mg l^−1^ hygromycin B and 35 mg l^−1^ G-418 (FUJIFILM Wako Pure Chemical) for proxy-CRISPR experiment.

### Heteroduplex mobility assay

The heteroduplex mobility assay (HMA) was used to detect targeted mutations induced by BlCas9, enBlCas9, or SpRY. One-month after transformation by *Agrobacterium*-mediated method, genomic DNA was extracted and the target loci were amplified by PCR using the primers shown in Supplementary Table S1 and KOD FX Neo DNA Polymerase (TOYOBO). The PCR products were diluted 15-fold with water, and analyzed by a microchip electrophoresis using MCE-202 MultiNA with a DNA-500 kit (SHIMADZU, Kyoto, Japan).

### Amplicon-sequencing analysis

For the analysis of targeted mutation frequencies, four callus samples were selected from HMA-analyzed lines and subjected to amplicon sequencing. For each target site, four transgenic callus lines were selected based on the HMA results. When fewer than four lines showed band shifts in HMA, a total of four calli were randomly chosen from the transgenic lines. For the analysis of base-editing frequencies, approximately 40 independent transgenic callus lines were obtained for each target site, and amplicon sequencing was performed for all transgenic lines. Genomic DNA was extracted from transgenic calli cultured for 1 month. Target loci were amplified by PCR using primers shown in Supplementary Table S1 and PrimeSTAR GXL DNA Polymerase (Takara Bio). PCR products were purified using Agencourt AMPure XP (Beckman Coulter, CA, USA) and used as templates for a 2nd round of PCR to attach sequence adapters IDT for Illumina DNA/RNA UD indexes (Illumina, San Diego, CA, USA) for amplicon-sequencing libraries. The 2nd round PCR products were purified using Agencourt AMPure XP, fragment sizes were checked by agarose gel electrophoresis, and the concentration measured using Qubit 2.0 Fluorometer and Qubit dsDNA HS Assay Kits (Thermo Fisher Scientific). The sequencing libraries were subjected to paired-end sequencing on a Miseq sequencer (Illumina). NGS data were analyzed using the software CRISPResso2 version 2.2.14 (Clement et al. 2019). The datasets presented in this study can be obtained from the corresponding author upon reasonable request.

## Results

### enBlCas9 can broaden the target scope, allowing more targeted mutations in rice

**Figure 2:**
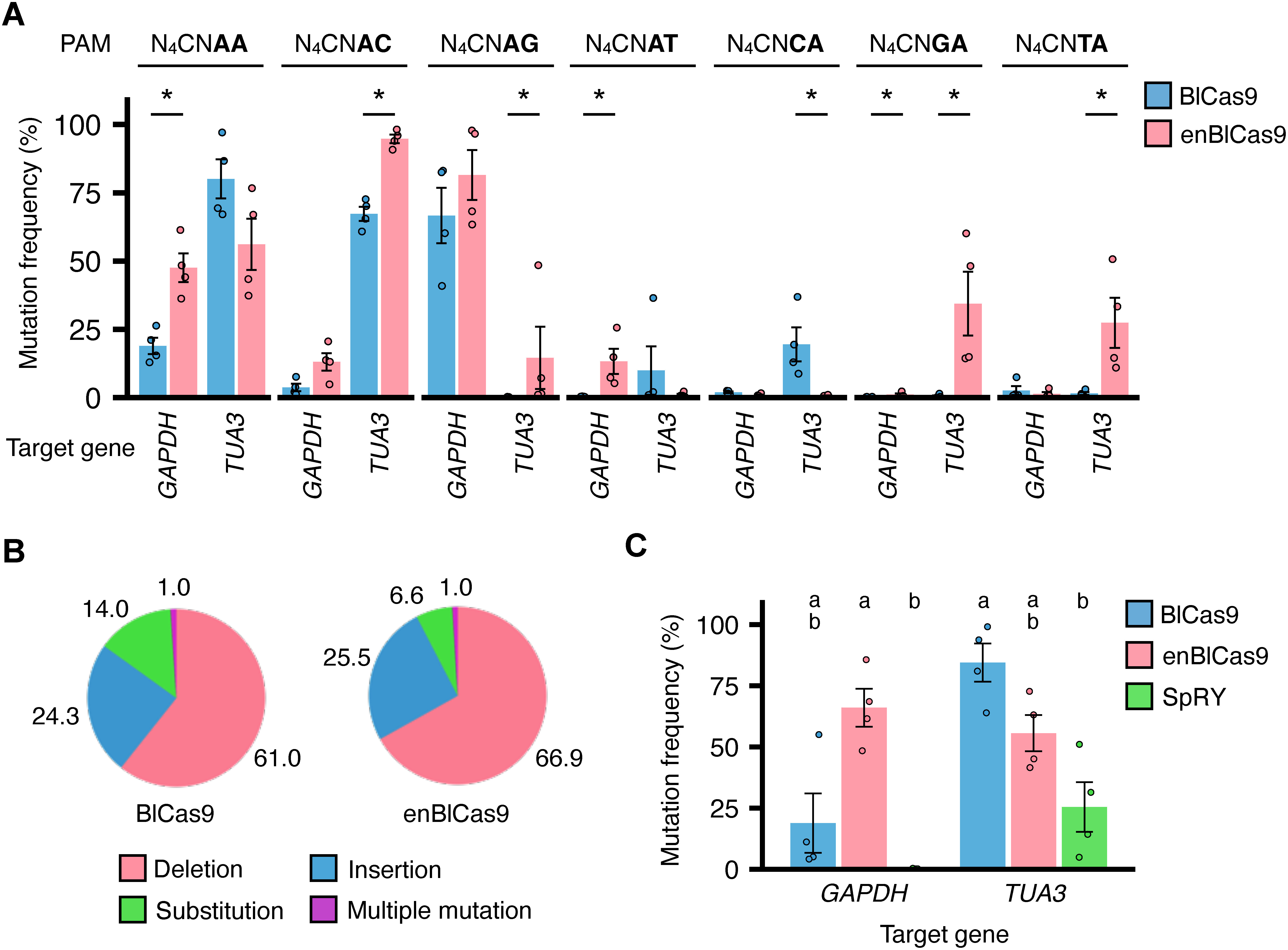
Figure 2. Targeted mutagenesis using BlCas9 and enBlCas9 in rice. (A) Mutation frequencies at 14 target sites using BlCas9 and enBlCas9. Dots represent mutation frequencies for each of four independent calli (*n*=4). Bars show the average mutation frequency, with error bars indicating the standard error. An asterisk denotes a statistically significant difference (*p*<0.05) between BlCas9 and enBlCas9 (Wilcoxon rank sum test). (B) Mutation patterns detected in BlCas9- and enBlCas9-mediated targeted mutagenesis. (C) Mutation frequencies using BlCas9, enBlCas9, and SpRY. Dots represent mutation frequencies for each callus line (*n*=4). Bars show the average mutation frequency of four independent calli, with error bars indicating the standard error. Different letters indicate statistically significant differences at *p*<0.05 (Kruskal–Wallis+Dunn’s test with FDR).

To investigate BlCas9- and enBlCas9-mediated targeted mutagenesis in rice, BlCas9/enBlCas9 and the corresponding sgRNA expression binary vectors for rice transformation were constructed (Figure 1A). Since previous studies have reported that BlCas9 shows a strong preference in vivo for a C at the 5th position of the PAM and a pronounced preference for an A at the 7th and 8th positions (Gao et al. 2020; Karvelis et al. 2015; Nakane et al. 2024), we designed 14 target sites in the *GLYCERALDEHYDE 3-PHOSPHATE DEHYDROGENASE* (*GAPDH*) and *TUBULIN ALPHA-3 CHAIN* (*TUA3*) genes with N_4_CNAN and N_4_CNNA PAMs (Supplementary Table S2) and tested whether enBlCas9 can induce mutations beyond the scope of the original PAM limitation of BlCas9 in rice. The genomic DNA was extracted from the transgenic calli one month after transformation. The target locus was amplified from the genomic DNA of each transgenic callus, and PCR products were subjected to HMA (Supplementary Figure S1), and the mutation frequencies of each callus were examined by amplicon-sequencing analysis (Figure 2A). When using BlCas9, the mutation frequencies were 19% and 80% at target sites with A at the 7th and 8th positions of the PAM, respectively. Of the 12 target sites with a non-A at the 7th or 8th positions of the PAM, only three target sites (N_4_CNAG at *GAPDH*, N_4_CNAC and N_4_CNCA at *TUA3*) showed mutation frequencies higher than 10% (Figure 2A). In contrast, the average mutation frequencies of enBlCas9 were significantly higher than that of BlCas9 at 7 of 14 target sites.

We compared the mutation pattern between BlCas9 and enBlCas9 (Figure 2B). Using either BlCas9 or enBlCas9, mainly small deletion or insertion mutations were detected, suggesting that structure-based enBlCas9 engineering did not change the mutation pattern. These results indicate that enBlCas9 can broaden the target scope and improve the frequency of targeted mutation in rice.

SpRY has been developed as a nearly PAM-less SpCas9 variant (Walton et al. 2020) and several groups have proposed its use for targeted mutagenesis and base editing in plants (Li et al. 2021; Ren et al. 2021; Xu et al. 2021). However, since the genome editing activity of SpRY at NYN (Y is C or T) PAM sites is lower than that at NRN (R is A or G) PAM sites, there are still only a few examples of efficient genome editing at NYN PAM target sites in plants. To compare the mutation efficiency at target sites where C is present in the PAM between BlCas9 variants and SpRY, we constructed a SpRY expression vector (Figure 1A), and selected SpRY targets where the 5th position C of the BlCas9 PAM is the 2nd position of the SpRY PAM (Supplementary Figure S2A). HMA and amplicon-sequencing analysis performed for investigating the mutation frequencies of BlCas9, enBlCas9, and SpRY (Figure 2C, Supplementary Figure S2B). The mutation frequency of enBlCas9 at the target site of the *GAPDH* gene and that of BlCas9 at the target site of the *TUA3* gene were significantly higher than those of SpRY, respectively (Figure 2C). These results suggest that, despite the limitations of the PAM sequence, the option to utilize BlCas9 and enBlCas9 in addition to SpRY can represent a strategy to enhance the mutation efficiency near C.

### Precise base substitutions using BlCas9 and enBlCas9

**Figure 3:**
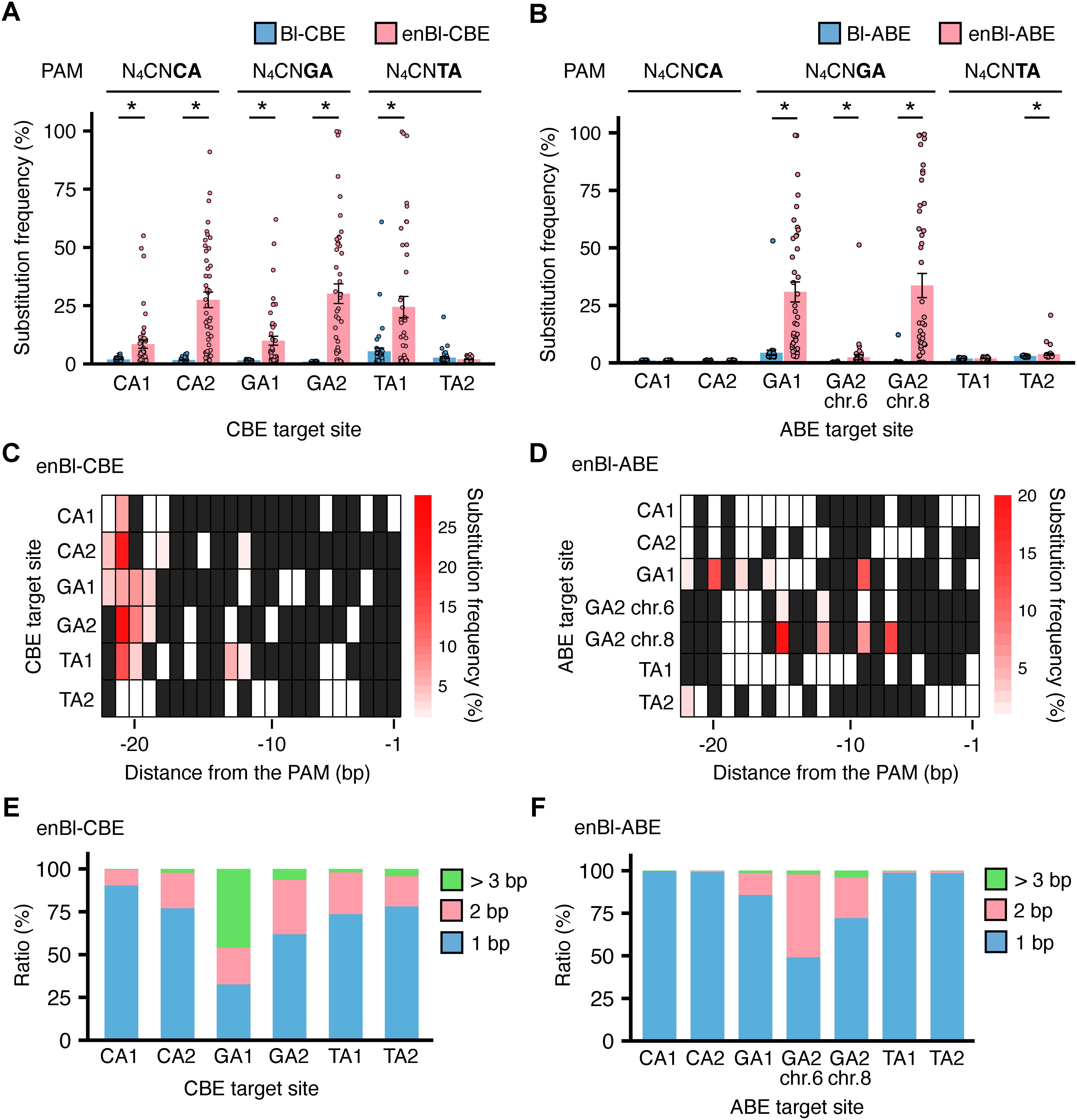
Figure 3. Cytosine and adenine base editing using BlCas9 and enBlCas9 in rice. (A) Substitution frequencies of Bl-CBE and enBl-CBE. Bars show the average substitution frequency of independent calli (*n*=42–48). Dots represent individual calli. Error bars indicate standard error. An asterisk denotes a statistically significant difference (*p*<0.05) between Bl-CBE and enBl-CBE (Wilcoxon rank sum test). (B) Substitution frequencies of Bl-ABE and enBl-ABE. Bars show the average substitution frequency of independent calli (*n*=37–46). Dots represent individual calli. Error bars indicate standard error. An asterisk denotes a statistically significant difference (*p*<0.05) between Bl-ABE and enBl-ABE (Wilcoxon rank sum test). (C) C-to-T substitution frequencies at each cytosine position across 6 target sites using enBl-CBE. Heatmap from white to red indicates average substitution frequency at each position. Gray areas represent bases other than cytosine. (D) A-to-G substitution frequencies at each adenine position across 7 target sites using enBl-ABE. Heatmap from white to red indicates average substitution frequency at each position. Gray areas represent bases other than adenine. (E) Distribution of substitution counts among sequences with C-to-T conversions mediated by enBl-CBE. (F) Distribution of substitution counts among sequences with A-to-G conversions mediated by enBl-ABE.

To introduce precise base editing in rice with an expanded target scope, we developed BlCas9- or enBlCas9-based CBEs and ABEs. The BlCas9- or enBlCas9-based CBEs (Bl-CBE/enBl-CBE) were derived from the target-activation-induced cytidine deaminase (target-AID) system, which utilizes a fusion of cytidine deaminase and uracil DNA glycosylase inhibitor to nCas9 (Figure 1B). In contrast, BlCas9-based ABE (Bl-ABE) and enBlCas9-based ABE (enBl-ABE) consist of adenosine deaminase ABE8e and nCas9 (Figure 1C). From the results of targeted mutation experiments (Figure 2A), these base editors were expected to be able to extend the targeting range to target sites where the 7th position of the PAM is not an A. Then, total 12 sgRNAs were designed for CBE and ABE target sites to examine base editing activity with a non-A at the 7th position of the PAM sequence (Supplementary Table S3). The genome DNA was extracted from transformed calli and mutations were detected by amplicon-sequencing analysis. Using amplicon-sequencing data, we investigated the substitution frequencies of BlCas9- (Figure 3A) and enBlCas9-based base editors (Figure 3B), the substitution positions in enBl-CBE (Figure 3C) and enBl-ABE (Figure 3D), and the tendencies of simultaneous multi-base substitutions in enBl-CBE (Figure 3E) and enBl-ABE (Figure 3F).

In the CBE experiments, the base substitution frequencies of enBl-CBE were significantly higher than those of Bl-CBE at five of the six target sites examined (Figure 3A). A few undesired mutations such as small deletions or insertions were detected at the target sites in both Bl-CBE and enBl-CBE expressing calli, although their frequencies were lower than that of precise C-to-T substitutions (Supplementary Figure S3A). We also analyzed off-target mutations of sgRNA CBE_CA2 and CBE_GA1 at off-target candidate sites with 1-nt mismatches in the target sequence and the 5th position of the PAM, respectively. The off-target mutation frequencies of CBE_CA2 and CBE_GA1 in enBl-CBE were less than 1.5% and were significantly lower than that of on-target sites (Supplementary Figure S4A, B). These results indicate that enBl-CBE can introduce precise C-to-T substitutions efficiently at target sites with relaxed PAM compared to Bl-CBE. The substitution frequency at each C in the target sequence indicated that the C-to-T substitutions were introduced mainly around −20 bp upstream of the PAM sequence for enBl-CBE (Figure 3C). The frequency of C-to-T substitutions in each sequence demonstrated that CBE_GA1 exhibited a high frequency of multiple substitutions, while the other targets exhibited single or double nucleotide substitutions primarily (Figure 3E). The top five most frequent base substitution patterns for each target sites were displayed in Supplementary Table S4. In CBE_GA1, a total of four mutation patterns were identified with a frequency greater than 10%. These patterns included C-to-T substitutions occurring within the region from −22 to −19 bp upstream of the PAM sequence. Furthermore, C-to-T substitutions in closer proximity to the PAM were detected without CBE_GA1 target sites. These results suggest that enBl-CBE could provide the useful tools to generate a wide variety of mutants.

In experiments using Bl-ABE and enBl-ABE, the ABE_GA2 sgRNA was designed to target two on-target sites in the rice genome, located on chromosomes 6 and 8 (Supplementary Table S3). Consequently, the substitution frequencies were investigated for a total of seven genomic loci, and enBl-ABE showed significantly higher substitution frequencies than Bl-ABE at four target sites (Figure 3B). However, the substitution frequency of enBl-ABE was comparable to that of Bl-ABE for the remaining targets. The frequency of undesired mutations was also measured in Bl-ABE and enBl-ABE, and they were rarely detected (Supplementary Figure S3B). These results suggest that enBl-ABE is more effective than Bl-ABE for precise A-to-G substitutions at a broader range in the rice genome. The substitution positions results observed in the ABE_GA1, which had high substitution frequencies, indicated that ABE_GA1 exhibited a high substitution frequency at the −20 and −9 bp upstream of the PAM sequence (Figure 3D). In contrast, in ABE_GA2-chr.8, the A-to-G substitutions were detected at A at the −15 to −7 bp. In the enBl-ABE experiments, the majority of A-to-G substitutions consisted of single- or double-base-pair changes (Figure 3F). Although the most prevalent mutation pattern was approximately 40% in both target sites, various A-to-G substitution patterns were detected (Supplementary Table S5). These results suggest that, like enBl-CBE, enBl-ABE also has the potential to facilitate the generation of a diversity of mutations.

### Proxy-CRISPR can improve BlCas9- and enBlCas9-mediated genome editing efficiency

**Figure 4:**
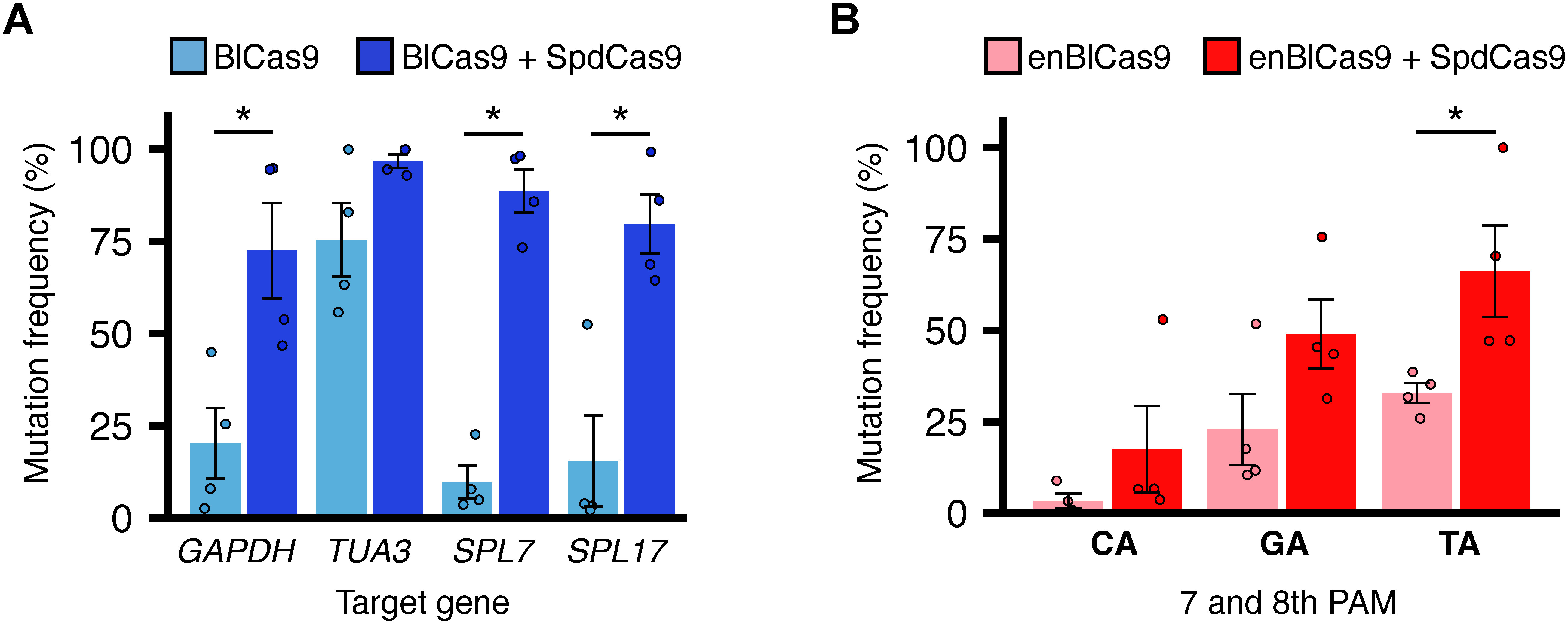
Figure 4. Proxy-CRISPR assisted genome editing using the BlCas9 and enBlCas9. (A) Mutation frequencies at 4 target sites using BlCas9 with or without SpdCas9. Dots represent mutation frequencies for individual calli (*n*=4). Bars show the average mutation frequency, with error bars indicating standard error. An asterisk denotes a statistically significant difference (*p*<0.05) between conditions with and without SpdCas9 (Wilcoxon rank sum test). (B) Mutation frequencies at three target sites using enBlCas9 with or without SpdCas9, where position 7 of the PAM contains C, G, or T. Dots represent mutation frequencies for individual calli (*n*=4). Bars show the average mutation frequency, with error bars indicating standard error. An asterisk denotes a statistically significant difference (*p*<0.05) between conditions with and without SpdCas9 (Wilcoxon rank sum test).

To improve the efficiency of genome editing with BlCas9 and enBlCas9, we focused on proxy-CRISPR, a strategy in which one or two dCas9 molecules are bound near the target sites of Cas orthologs (Chen et al. 2017). To investigate the proxy-CRISPR effect on BlCas9-mediated genome editing in rice, we constructed SpdCas9 expression vector (Figure 1D). The BlCas9 and SpdCas9 expression vectors were co-transformed into rice calli, and transformants were selected by both hygromycin and G-418 resistant phenotypes. As a previous study demonstrated that proxy-CRISPR is more effective when SpdCas9 target sites are placed both upstream and downstream of the target site (Chen et al. 2017), we first designed SpdCas9 sgRNAs flanking the four BlCas9 target site in *GAPDH*, *TUA3*, *SQUAMOSA PROMOTER BINDING PROTEIN- LIKE 7* (*SPL7*) and *SPL17* genes to evaluate the effect of proxy-CRISPR on BlCas9 activity (Supplementary Table S6). The target loci were amplified from the genome DNA of transgenic calli, and PCR products were subjected to HMA (Supplementary Figure S5A), and the mutation frequencies were examined by amplicon sequencing analysis. Co-expression of SpdCas9 and its sgRNAs with BlCas9 resulted in the increase average mutation frequency across all tested target sites. Among these targets, three targets demonstrated a significant increase in mutation frequency when compared to the use of BlCas9 alone (Figure 4A). We then investigated whether proxy-CRISPR is also effective for enBlCas9-mediated genome editing at expanded target sites. SpdCas9 target sites were designed around three enBlCas9 target sites in the *TUA3* gene, which contain C, G, or T at position 7 of their PAM, respectively (Supplementary Table S6). A significant increase in mutation frequencies was exclusively observed at the TA PAM target (Figure 4B). However, for all target sites, mutation frequencies were at least 2-fold higher when enBlCas9 was co-expressed with SpdCas9 than when enBlCas9 was used alone (Figure 4B, Supplementary Figure S5B). These results indicate that proxy-CRISPR enhances the mutation efficiency of enBlCas9 at expanded PAM target sites.

## Discussion

Genome editing technology is used to confer agriculturally useful traits to crops, such as herbicide resistance and enhanced grain yield (Hendelman et al. 2021; Liu et al. 2021; Zhang et al. 2021). These phenotypes are achieved not only by loss-of-function mutations but also by gain-of-function mutations based on amino acid substitutions or by adjusting gene expression levels. In CRISPR/Cas9-based genome editing tools, DNA cleavage and base substitution occur at a certain distance from the PAM sequence. Therefore, adding Cas options with different PAM sequences can increase the flexibility of genome editing. In this study, we showed that BlCas9 can induce mutations at several N_4_CNAN PAM target sites in rice. Mutation frequency varied depending on the target sequence, even when the 7th and 8th positions of the PAM sequence were the same, consistent with previous studies (Gao et al. 2020; Karvelis et al. 2015; Nakane et al. 2024). These results reveal that the mutation efficiency of BlCas9 is affected not only by PAM sequences but also by protospacer sequences and/or chromatin structure. Recent structure-based engineering has succeeded in enhancing the DNA cleavage activity of Cas9 orthologs (Nakagawa et al. 2022). Nakane and colleagues have developed a rationally designed enBlCas9 (Nakane et al. 2024).

In the present study, we revealed that enBlCas9 can recognize more relaxed PAM sequences and exhibits enhanced mutation activity in rice compared with BlCas9, consistent with results obtained in vitro and in human cells (Nakane et al. 2024). The genome editing frequencies of enBlCas9 were significantly higher than those of BlCas9 at 7 of the 14 target sites examined, suggesting that enBlCas9 has a broader PAM recognition range than BlCas9 (Figure 2A). However, at the TUA3 target site with an N_4_CNCA PAM, detectable mutations were observed only with BlCas9 (Figure 2A). Nakane et al. (2024) has reported that, in human cells, some target sites with an N_4_CNCA PAM were edited by BlCas9 but not by enBlCas9. In addition, genome editing by BlCas9 at PAMs containing a cytosine at the 7th or 8th position has also been reported (Gao et al. 2020). The higher editing frequency observed with BlCas9 at the TUA3 N_4_CNCA site is not inconsistent with previous findings. Together, these observations support the idea that, although PAM preference is a major determinant of Cas9 activity, target- and context-dependent factors can modulate genome editing efficiency in vivo.

For both BlCas9 and enBlCas9, the mutation patterns were dominated by deletions and insertions, and no major differences were observed between the two nucleases (Figure 2B). Although BlCas9 appears to show a higher proportion of base substitutions than enBlCas9, this is attributable to the markedly lower genome-editing efficiencies of BlCas9 at many of the analyzed target sites. As a result, inevitable background sequencing errors—most of which are interpreted as base substitutions—constitute a larger fraction of the total detected mutations when using BlCas9.

We also generated enBlCas9-based CBEs and ABEs which can introduce precise base substitutions at the expand target sites (Figure 3A, B). It was reported to SpCas9-based CBEs mainly introduce C-to-T substitutions within the range of three to five bases surrounding the −18 position upstream of the PAM sequence (Endo et al. 2019; Nishida et al. 2016), and A-to-G substitutions induced by SpCas9 variants-based ABEs are found −12 to −18 bp from PAM sequences (Negishi et al. 2019; Ren et al. 2021). In contrast, more widely range base substations were detected at several target sites in our enBlCas9-based base editors such as CBE_CA2, CBE_GA2, CBE_TA1, ABE_GA1 and ABE_GA2-chr.8 (Figure 3C, D, Supplementary Tables S4, S5). These results imply that enBlCas9-based CBE and ABE have a wide editing window. Previous studies have suggested that broader base-editing windows can be achieved when steric interference between the Cas protein and the deaminase is reduced (Hao et al. 2024). Such effects have been reported using Cas proteins other than SpCas9 (Hao et al. 2024; Zhang et al. 2020). Therefore, structural differences between BlCas9 and SpCas9, including the smaller size of BlCas9, may reduce steric constraints on the deaminase and contribute to the wider editing window observed with enBlCas9-based base editors.

As with other Cas9s, the mutational efficiency of BlCas9 varies widely among target sites, possibly due in part to differences in the chromatin structure of each target. Indeed, it was reported to the nucleosomes inhibit Cas9 target access and cleavage (Horlbeck et al. 2016) and the modulating chromatin accessibility enhances Cas9 editing efficiency in rice (Liu et al. 2019; Nagamura et al. 2024). Proxy-CRISPR can modify the local chromatin structures by SpdCas9 binding at the proximal location of the target sequence (Chen et al. 2017). In this study, we showed that the mutation efficiencies of BlCas9 and enBlCas9 were improved by proxy-CRISPR. According to the open chromatin map generated from DNase I sensitivity profiles in rice calli, the TUA3 target site—where only a limited proxy-CRISPR effect was observed—appears to reside within an open chromatin region. In contrast, the remaining three target sites, which exhibited substantial enhancement by the proxy-CRISPR strategy, were all located within regions predicted to be closed chromatin (Nagamura et al. 2024; Zhang et al. 2012). These observations suggest that BlCas9 tends to exhibit reduced mutagenesis efficiency in closed chromatin regions, and that proxy-CRISPR represents an effective approach to expand the range of BlCas9-editable sites in the rice genome. It was reported to proxy-CRISPR also improves base editing efficiency (Lian et al. 2021). Therefore, a combination of our enBlCas9-based genome editing technology and proxy-CRISPR could support the efficiency of precision genome editing in plants. The use of small-molecule Cas variants, such as *Acidibacillus sulfuroxidans* Cas12f (422 amino acids), has the advantage that they can be delivered into the plant cells using virus-derived vectors, allowing transgene-free genome editing (Ishibashi et al. 2024). Since the molecular size of BlCas9 (1,092 amino acids) is smaller than that of SpCas9 (1,368 amino acids), BlCas9 may be efficiently loaded into plant RNA virus vectors such as *potato virus X* and *sonchus yellow net rhabdovirus*, which have been used as carriers of genome editing tools for DNA-free genome editing (Ariga et al. 2020; Ma et al. 2020). In future studies, we expect that enBlCas9-based genome editing will greatly facilitate precise, transgene-free, genome editing in plants.

## Abbreviations

ABE: adenine base editor
ADH: ALCOHOL DEHYDROGENASE 5
AID: activation-induced cytidine deaminase
Bl-ABE: BlCas9-based adenine base editor
Bl-CBE: BlCas9-based cytosine base editor
bpNLS: bipartite nuclear localization signal
Cas: CRISPR-associated protein
CBE: cytosine base editor
CDA1: cytidine deaminase 1
CRISPR: clustered regularly interspaced short palindromic repeats
dCas9: catalytically dead Cas9
enBlCas9: enhanced BlCas9
enBl-ABE: enBlCas9-based adenine base editor
enBl-CBE: enBlCas9-based cytosine base editor
G-418: geneticin reagent
GAPDH: GLYCERALDEHYDE 3-PHOSPHATE DEHYDROGENASE
HMA: heteroduplex mobility assay
HPTII: hygromycin B phosphotransferase II
nCas9: Cas9 nickase
nos: nopaline synthase
NPT II: neomycin phosphotransferase II
PAM: protospacer adjacent motif
Proxy-CRISPR: proximal CRISPR targeting method
sgRNA: single guide RNA
SPL: SQUAMOSA PROMOTER BINDING PROTEIN-LIKE
TUA3: TUBULIN ALPHA-3 CHAIN
U6: U6-2 small nuclear RNA
UBI: UBIQUITIN 1
UGI: uracil DNA glycosylase inhibitor
UTR: untranslated region
35S: cauliflower mosaic virus 35S

## Description of supplementary files

Supplementary Figure S1. HMA of BlCas9 or enBlCas9 at *GAPDH* and *TUA3* target sites.

Supplementary Figure S2. Targeted mutagenesis using BlCas9, enBlCas9, and SpRY.

Supplementary Figure S3. Undesired mutation frequency of BlCas9- or enBlCas9-mediated base editors.

Supplementary Figure S4. Off-target mutation frequency of Bl-CBE and enBl-CBE.

Supplementary Figure S5. HMA of proxy-CRISPR experiments.

Supplementary Table S1. List of primers used in this study.

Supplementary Table S2. Target and PAM sequences for targeted mutagenesis.

Supplementary Table S3. Target and PAM sequences for base editing.

Supplementary Table S4. The substitution patterns introduced by enBl-CBE.

Supplementary Table S5. The substitution patterns introduced by enBl-ABE.

Supplementary Table S6. Target and PAM sequences, and distance from the BlCas9 target sites for proxy-CRISPR.
