# Supplementary material for: Targeted mutagenesis and base editing using engineered Brevibacillus laterosporus Cas9 with expanded target scope in rice
Source: Plant Biotechnol (Tokyo). 2026 Jun 25;43(2):151–60. doi: 10.5511/plantbiotechnology.26.0218a (PMC13324213; doi:10.5511/plantbiotechnology.26.0218a)
Supplement: Supplementary Data [file plantbiotechnology-43-2-26.0218a-s001.pdf]

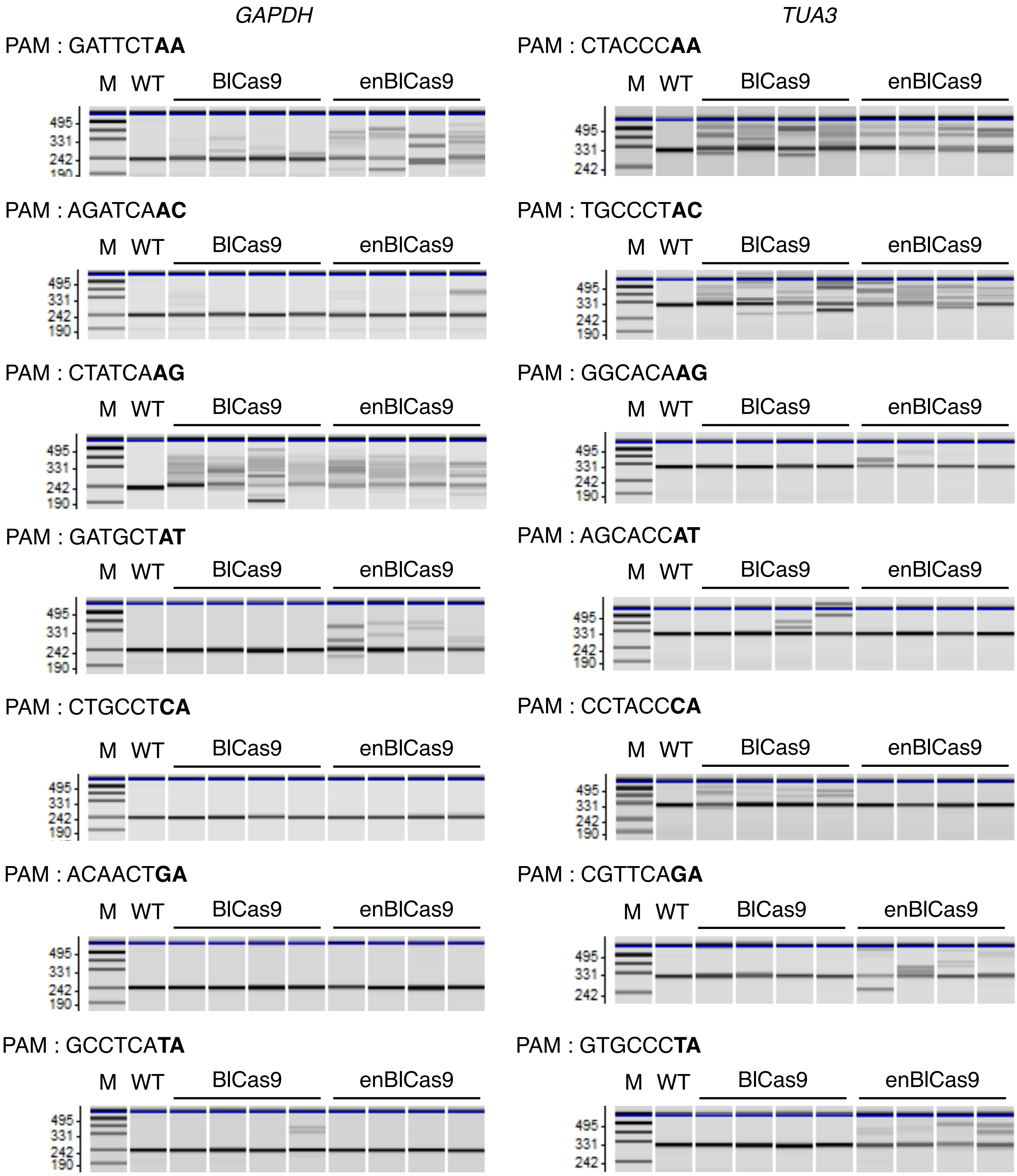

Supplementary Figure S1. HMA of BlCas9 or enBlCas9 at *GAPDH* and *TUA3* target sites.
M, DNA molecular weight marker; WT, wild-type (Nipponbare).

A

GAPDH

BlCas9 and enBlCas9 target sequence

CATCATATGAGGCAGCCTTCTC**GATTCTAA**

5'…ATAGCATCATATGAGGCAGCCTTCTCGATTCTAACTGT…3'

TATGAGGCAGCCTTCTCGAT**TCT**

SpRY target sequence

TUA3

BlCas9 and enBlCas9 target sequence

AGTTCCAGACCAACCTTGTGCC**CTACCCAA**

5'…AATGAGTTCCAGACCAACCTTGTGCCCTACCCAAGGAT…3'

CAGACCAACCTTGTGCCCTA**CCC**

SpRY target sequence

B

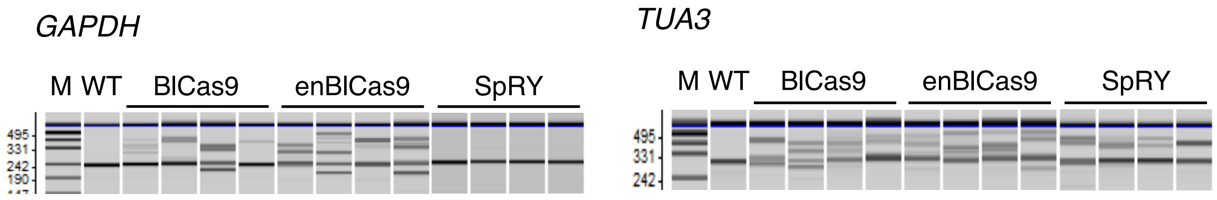

Supplementary Figure S2. Targeted mutagenesis using BlCas9, enBlCas9, and SpRY.

(A) Target sequences of BlCas9, enBlCas9, and SpRY. The blue characters mean PAM sequence for BlCas9 and enBlCas9. The green characters mean PAM sequence for SpRY. The red C in the genome sequence is the 5th position of the BlCas9 PAM and the 2nd position of the SpRY PAM.

(B) HMA of representative lines using BlCas9 or enBlCas9 at *GAPDH* and *TUA3* target sites. M, DNA molecular weight marker; WT, wild-type (Nipponbare).

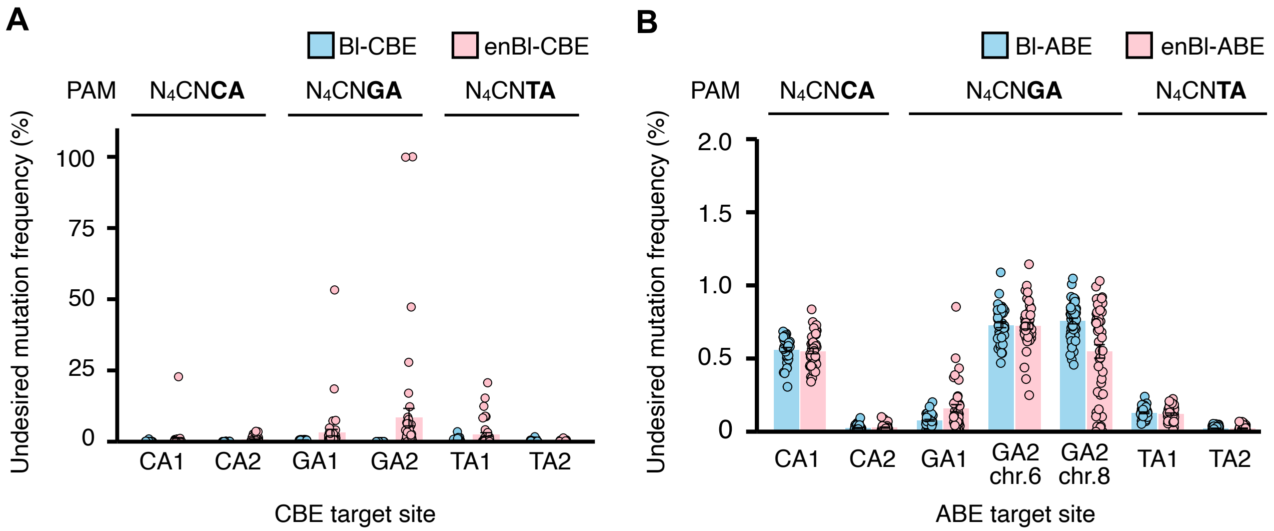

Supplementary Figure S3.Undesired mutation frequency of BlCas9- or enBlCas9-mediated base editors.

(A)Undesired mutation frequency of Bl-CBE and enBl-CBE.

Undesired means not-C-to-T substitution such as deletion, insertion, or other base substitutions. The error bars indicate standard error (n = 42 – 48).

(B)Undesired mutation frequency of Bl-ABE and enBl-ABE.

Undesired means not-A-to-G substitution such as deletion, insertion, or other base substitutions. The error bars indicate standard error (n = 37 – 46).

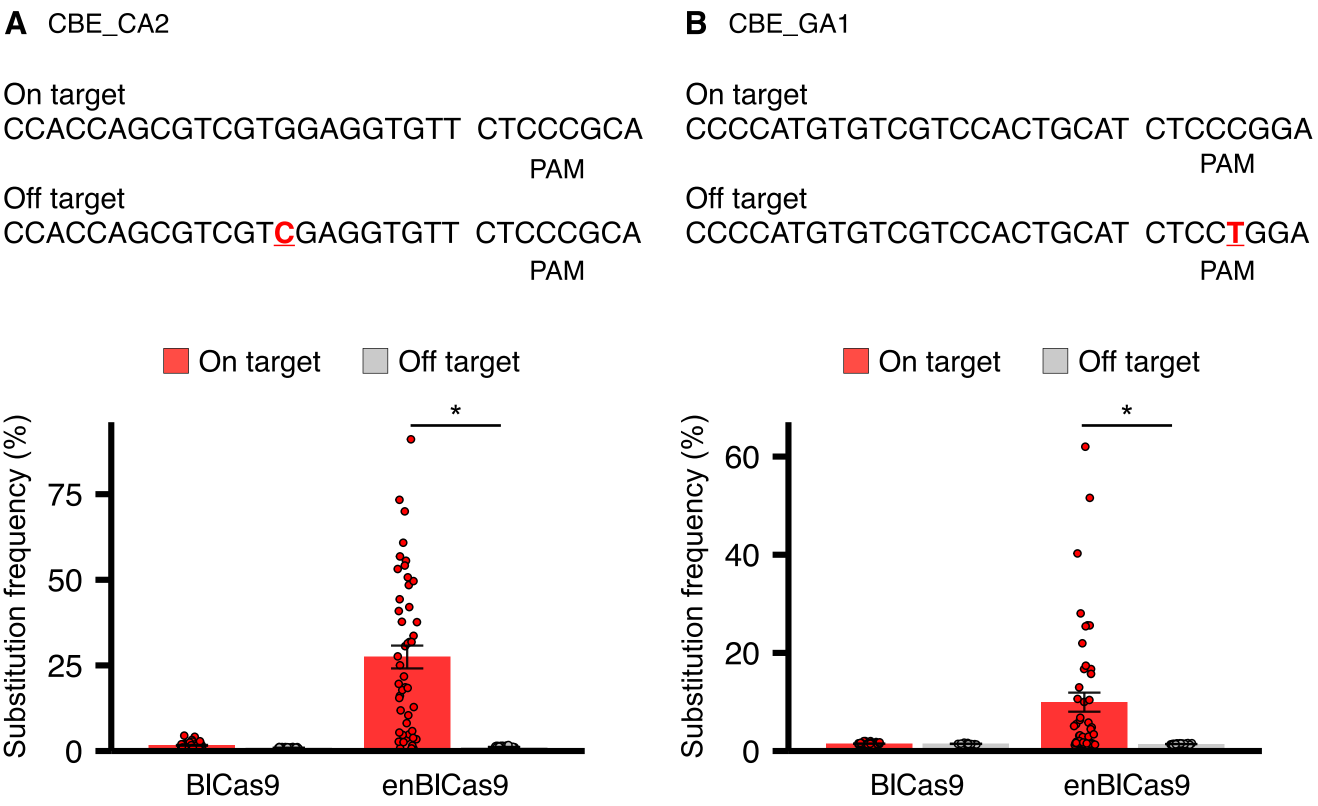

Supplementary Figure S4. Off-target mutation frequency of Bl-CBE and enBl-CBE.
(A)On-target indicates CBE_CA2 target sequence and the off-target site has a single mismatch at 9 bp upstream of the PAM. The error bars indicate standard error (n = 47 – 48). An asterisk indicates a significant difference at $P < 0.05$ between the substitution frequency of on target and off target (Wilcoxon rank sum test).
(B)On-target indicates CBE_GA1 target sequence and the off-target site has a single mismatch at the 5th position of the PAM. The error bars indicate standard error (n = 48). An asterisk indicates a significant difference at $P < 0.05$ between the substitution frequency of on target and off target (Wilcoxon rank sum test).

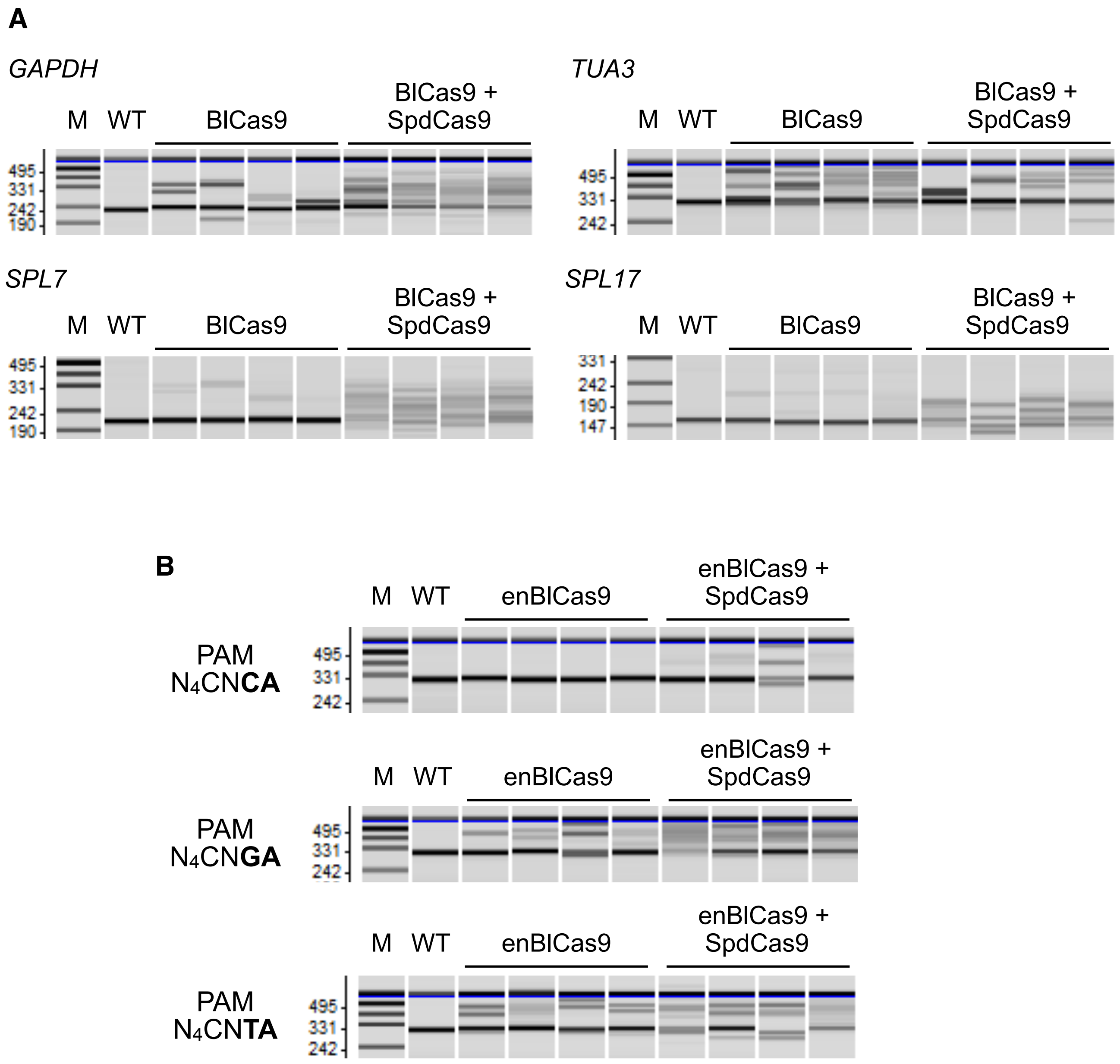

Supplementary Figure S5. HMA of proxy-CRISPR experiments.

(A)HMA of representative lines using BlCas9 with proxy-CRISPR at *GAPDH, TUA3, SPL7* and *SPL17* target sites. M, DNA molecular weight marker; WT, wild-type (Nipponbare).

(B)HMA of representative lines using enBlCas9 with proxy-CRISPR at N_4CNCA, N_4CNGA, and N_4CNTA PAM in *TUA3* target sites. M, DNA molecular weight marker; WT, wild-type (Nipponbare).

Supplementary Table S1. List of primers used in this study.

Primer name	Sequence
GAPDH-HMA_Fw	CTGCCACAAGATGCAGTTCTAG
GAPDH-HMA_Rv	TCAATGCATTGCAGGCTGTTGGC
GAPDH-amplicon_Fw	TCGTCGGCAGCGTCAGATGTGTATAAGAGACAGGCATGTTTTGTTAGTAAAATGGGACTGCAGCC
GAPDH-amplicon_Rv	GTCTCGTGGGCTCGGAGATGTGTATAAGAGACAGCCAACACACGTAACTACACTTTTCTAGGAATCC
TUA3-HMA_Fw	ACAGGCTTGTGTCTCAGGTACAGTCT
TUA3-HMA_Rv	AGGCACTGTTGGTGATCTCGGCAACAGA
TUA3-amplicon_Fw	TCGTCGGCAGCGTCAGATGTGTATAAGAGACAGCAGTCTTGTATCCTTGCAATGATCTCAAATTCTCTG
TUA3-amplicon_Rv	GTCTCGTGGGCTCGGAGATGTGTATAAGAGACAGCACGACATCGCCGCGGTACATGAG
CBE_CA1-amplicon_Fw	GTCTCGTGGGCTCGGAGATGTGTATAAGAGACAGAGGCTGTCTGGCCCTATAAAACGG
CBE_CA1-amplicon_Rv	TCGTCGGCAGCGTCAGATGTGTATAAGAGACAGACATCCAACAACCGCGAATCCACC
CBE_CA2-amplicon_Fw	GTCTCGTGGGCTCGGAGATGTGTATAAGAGACAGAAGCGCACCATCCAGTTCGTGG
CBE_CA2-amplicon_Rv	TCGTCGGCAGCGTCAGATGTGTATAAGAGACAGACCTCCTCGTAGTCCTTCTCCA
CBE_CA2_off-target-amplicon_Fw	GTCTCGTGGGCTCGGAGATGTGTATAAGAGACAGAAGCGCACCATCCAGTTCGTGG
CBE_CA2_off-target-amplicon_Rv	TCGTCGGCAGCGTCAGATGTGTATAAGAGACAGACCTCCTCGTAGTCCTTCTCCA
CBE_GA1-amplicon_Fw	GTCTCGTGGGCTCGGAGATGTGTATAAGAGACAGAGACTTGATGCATCACATTGGTAGACAC
CBE_GA1-amplicon_Rv	TCGTCGGCAGCGTCAGATGTGTATAAGAGACAGTTCAGATTCTGCTTGGCGTGACG
CBE_GA1_off-target-amplicon_Fw	GTCTCGTGGGCTCGGAGATGTGTATAAGAGACAGGAAGACTTGATGCATCACATTGGTAGAC
CBE_GA1_off-target-amplicon_Rv	TCGTCGGCAGCGTCAGATGTGTATAAGAGACAGCCCAATCTCTGCTGCTAGTTCTTCC
CBE_GA2-amplicon_Fw	GTCTCGTGGGCTCGGAGATGTGTATAAGAGACAGCTTATGAAAAACTCTTTCTTGCATATAGAC
CBE_GA2-amplicon_Rv	TCGTCGGCAGCGTCAGATGTGTATAAGAGACAGACGCGTACGATTGGTCGTATGTG
CBE_TA1-amplicon_Fw	GTCTCGTGGGCTCGGAGATGTGTATAAGAGACAGACACCATGTTCATGACAGCATCGACC
CBE_TA1-amplicon_Rv	TCGTCGGCAGCGTCAGATGTGTATAAGAGACAGTCGAGCATGGCATCCAGGTACGG
CBE_TA2-amplicon_Fw	GTCTCGTGGGCTCGGAGATGTGTATAAGAGACAGTGAGCTCTACCATGTGTCGTAGTTGG
CBE_TA2-amplicon_Rv	TCGTCGGCAGCGTCAGATGTGTATAAGAGACAGGGAATATCAGTGACCAACCTGTGGAC
ABE_CA1-amplicon_Fw	GTCTCGTGGGCTCGGAGATGTGTATAAGAGACAGTGAGCTAAAACCACTGTAGAACCCTAC
ABE_CA1-amplicon_Rv	TCGTCGGCAGCGTCAGATGTGTATAAGAGACAGGGTGAATTTTCTGGTGGTGTTTTTGG
ABE_CA2-amplicon_Fw	GTCTCGTGGGCTCGGAGATGTGTATAAGAGACAGTCATCACCTCCACCAACAGTCCTG
ABE_CA2-amplicon_Rv	TCGTCGGCAGCGTCAGATGTGTATAAGAGACAGCATATGTCTAGTGTTTGGTGACACTGC
ABE_GA1-amplicon_Fw	GTCTCGTGGGCTCGGAGATGTGTATAAGAGACAGACACCATGTTCATGACAGCATCGACC
ABE_GA1-amplicon_Rv	TCGTCGGCAGCGTCAGATGTGTATAAGAGACAGTCGAGCATGGCATCCAGGTACGG
ABE_GA2-amplicon_Fw	GTCTCGTGGGCTCGGAGATGTGTATAAGAGACAGTTATCACAACTGAACCAAGACTCATTCC
ABE_GA2-amplicon_Rv	TCGTCGGCAGCGTCAGATGTGTATAAGAGACAGGCTTTGTATTGGGCTCAGTATGTAGAG
ABE_TA1-amplicon_Fw	GTCTCGTGGGCTCGGAGATGTGTATAAGAGACAGAACAACTCTGAACAGAAGCATGAACAGC
ABE_TA1-amplicon_Rv	TCGTCGGCAGCGTCAGATGTGTATAAGAGACAGAGGGCGGCCAGTTTTAACATCATTCC
ABE_TA2-amplicon_Fw	GTCTCGTGGGCTCGGAGATGTGTATAAGAGACAGTCATCACCTCCACCAACAGTCCTG
ABE_TA2-amplicon_Rv	TCGTCGGCAGCGTCAGATGTGTATAAGAGACAGCATATGTCTAGTGTTTGGTGACACTGC
SPL7-HMA_Fw	TCAGGGGAAGAGGAAGGAGAAG
SPL7-HMA_Rv	GTAGATCGTGGTGATTGACGCAC
SPL7-amplicon_Fw	TCGTCGGCAGCGTCAGATGTGTATAAGAGACAGTGGTGGTTCCGCGGATCAGGGGAA
SPL7-amplicon_Rv	GTCTCGTGGGCTCGGAGATGTGTATAAGAGACAGGGCAGCTCTTCTTGGCGTCGTCAAAC
SPL17-HMA_Fw	AGGTGCCAGGTGGAGGGTTG
SPL17-HMA_Rv	GGTTTTTTACCTGCTGCACTGTTGG
SPL17-amplicon_Fw	TCGTCGGCAGCGTCAGATGTGTATAAGAGACAGTTCGGCAAGAAGATCTACTTCGAGCAGGA
SPL17-amplicon_Rv	GTCTCGTGGGCTCGGAGATGTGTATAAGAGACAGACGAGGAGGAGGAAGAAGAAGAAGATTTTGG

Supplementary Table S2. Target and PAM sequences for targeted mutagenesis.

PAM	Target gene	Target sequence	PAM sequence
N_4CN**AA**	*GAPDH*	CATCATATGAGGCAGCCTTCTC	GATTCTAA
	TUA3	AGTTCCAGACCAACCTTGTGCC	CTACCCAA
N_4CN**AC**	*GAPDH*	AGCCTTCTCGATTCTAACTGTG	AGATCAAC
	TUA3	AATGAGTTCCAGACCAACCTTG	TGCCCTAC
N_4CN**AG**	*GAPDH*	GAGAAGGCTGCCTCATATGATG	CTATCAAG
	TUA3	CATGAAGTGGATCCTTGGGTAG	GGCACAAG
N_4CN**AT**	*GAPDH*	AATCGAGAAGGCTGCCTCATAT	GATGCTAT
	TUA3	ACTCATTGACATCCACGTTCAG	AGCACCAT
N_4CN**CA**	*GAPDH*	CTCACAGTTAGAATCGAGAAGG	CTGCCTCA
	TUA3	GAGTTCCAGACCAACCTTGTGC	CCTACCCA
N_4CN**GA**	*GAPDH*	CTCGATTCTAACTGTGAGATCA	ACAACTGA
	TUA3	GTCTGGAACTCATTGACATCCA	CGTTCAGA
N_4CN**TA**	*GAPDH*	CACAGTTAGAATCGAGAAGGCT	GCCTCATA
	TUA3	CAATGAGTTCCAGACCAACCTT	GTGCCCTA

Supplementary Table S3. Target and PAM sequences for base editing.

Base editor	PAM	Target site	Target sequence	PAM sequence
CBE	N_4CN**CA**	CBE_CA1	CCGCCATGAGGGAGTGCATCTC	GATCCACA
		CBE_CA2	CCACCAGCGTCGTGGAGGTGTT	CTCCCGCA
	N_4CN**GA**	CBE_GA1	CCCCATGTGTCGTCCACTGCAT	CTCCCGGA
		CBE_GA2	TCCCTGCAACAGCATTGGCTGA	ATGCCTGA
	N_4CN**TA**	CBE_TA1	TCCACGCATCCTAGGACCAGAT	CCACCATA
		CBE_TA2	ACCCAGATGCACCAAGCCCAAG	TGACCCTA
ABE	N_4CN**CA**	ABE_CA1	ACACAAAAAACCCCCACTCAAA	TCACCACA
		ABE_CA2	AGATAACACAATTCAAAAGACT	GACCCACA
	N_4CN**GA**	ABE_GA1	ACAGAGAAAACGCAGATCCAGA	CAACCAGA
		ABE_GA2 chr.6	CTGAAATACCACCACACTTCCG	TTGCCTGA
		ABE_GA2 chr.8	CTGAAATACCACCACACTTCCG	CTGCCTGA
	N_4CN**TA**	ABE_TA1	GTCAAAAAGAAAACTATACGCA	GCCCCATA
		ABE_TA2	AAGATACAAGATCGTTTCAAAA	CTACCATA

Supplementary Table S4. The substitution patterns introduced by enBI-CBE.

CBE target	Mutation sequence	Ratio (%)	Substitution positions (bp)
CA1	CTGCCATGAGGGAGTGCATCTC	83.4	-21
	CTGTCATGAGGGAGTGCATCTC	7.3	-21, -19
	CCGCCATGAGGGAGTGCATTTC	4.2	-3
	TTGCCATGAGGGAGTGCATCTC	1.8	-22, -21
	CCGTCATGAGGGAGTGCATCTC	0.8	-19
CA2	CTACCAGCGTCGTGGAGGTGTT	66.5	-21
	TTACCAGCGTCGTGGAGGTGTT	11.0	-22, -21
	TCACCAGCGTCGTGGAGGTGTT	7.6	-22
	CTACCAGCGTTGTGGAGGTGTT	5.2	-21, -12
	CTACTAGCGTCGTGGAGGTGTT	3.5	-21, -18
GA1	CCTCATGTGTCGTCCACTGCAT	24.6	-20
	TTTTATGTGTCGTCCACTGCAT	23.3	-22, -21, -20, -19
	CTTCATGTGTCGTCCACTGCAT	15.5	-21, -20
	TTTCATGTGTCGTCCACTGCAT	11.9	-22, -21, -20
	CTTTATGTGTCGTCCACTGCAT	7.4	-21, -20, -19
GA2	TTCCTGCAACAGCATTGGCTGA	61.0	-21
	TTTCTGCAACAGCATTGGCTGA	26.1	-21, -20
	TTCTTGCAACAGCATTGGCTGA	5.2	-21, -19
	TTTTTGCAACAGCATTGGCTGA	5.1	-21, -20, -19
	TTTCTGCAACAGTATTGGCTGA	0.5	-21, -20, -10
TA1	TTCACGCATCCTAGGACCAGAT	62.4	-21
	TTTACGCATCCTAGGACCAGAT	7.3	-21, -20
	TCCACGCATTCTAGGACCAGAT	7.2	-13
	TCCACGCATTTTAGGACCAGAT	6.4	-13, -12
	TCTACGCATTCTAGGACCAGAT	6.1	-20, -13
TA2	ACCCAGATGCACTAAGCCCAAG	40.9	-10
	ACTCAGATGCACTAAGCCCAAG	12.6	-20, -10
	ACTCAGATGCACCAAGCCCAAG	10.7	-20
	ACCCAGATGCACCAAGTCCAAG	4.7	-6
	ACCCAGATGCATCAAGCCCAAG	4.5	-11

Supplementary Table S5. The substitution patterns introduced by enBI-ABE.

ABE target	Mutation sequence	Ratio (%)	Substitution positions (bp)
	ACAC**G**AAAAACCCCCACTCAAA	15.6	-18
	ACACAAAAAACCCCCACTC**G**AA	11.6	-3
CA1	ACACA**G**AAAACCCCCACTCAAA	9.0	-17
	ACACAA**G**AAACCCCCACTCAAA	8.6	-16
	AC**G**CAAAAAACCCCCACTCAAA	8.4	-20
	AGATAACACAATTCAAA**G**GACT	26.3	-5
	AGATAAC**G**CAATTCAAAAGACT	11.1	-15
CA2	AGATAACAC**G**ATTCAAAAGACT	11.0	-13
	AGAT**G**ACACAATTCAAAAGACT	10.4	-18
	AGATAACACAATTC**G**AAAGACT	7.2	-8
	AC**G**GAGAAAACGCAGATCCAGA	38.8	-20
	ACAGAGAAAACGC**G**GATCCAGA	29.0	-9
GA1	ACAG**G**GAAAACGCAGATCCAGA	6.7	-18
	AC**G**GAGAAAACGC**G**GATCCAGA	5.3	-20, -9
	**G**CAGAGAAAACGCAGATCCAGA	3.8	-22
	CTGAAAT**G**CC**G**CCACACTTCCG	42.0	-15, -12
	CTGAAAT**G**CCACCACACTTCCG	32.0	-15
GA2 chr.6	CTGAAATACCACCAC**G**CTTCCG	5.9	-7
	CTGAAATACCACC**G**CACTTCCG	4.3	-9
	CTGAAATACC**G**CCACACTTCCG	3.9	-12
	CTGAAAT**G**CCACCACACTTCCG	42.7	-15
	CTGAAATACCACCAC**G**CTTCCG	14.8	-7
GA2 chr.8	CTGAAAT**G**CCACCAC**G**CTTCCG	11.9	-15, -7
	CTGAAATACCACC**G**CACTTCCG	8.5	-9
	CTGAAATACC**G**CCACACTTCCG	5.8	-12
	GTCAAAAAGAAAACT**G**TACGCA	16.5	-7
	GTC**G**AAAAGAAAACTATACGCA	13.7	-19
TA1	GTCAAAAAGAAAACTATACGC**G**	9.2	-1
	GTCAAAA**G**GAAAACTATACGCA	8.9	-15
	GTCAA**G**AAGAAAACTATACGCA	7.6	-17
	**G**AGATACAAGATCGTTTCAAAA	62.6	-22
	AAGAT**G**CAAGATCGTTTCAAAA	23.6	-17
TA2	AAGATACAAG**G**TCGTTTCAAAA	5.0	-12
	AAGATAC**G**AGATCGTTTCAAAA	2.0	-15
	AAGATACAAGATCGTTTC**G**AAA	1.2	-4

Supplementary Table S6. Target and PAM sequences, and distance from the BlCas9 target sites for proxy-CRISPR.

Cas9	PAM	Target gene	Target sequence	PAM sequence	Distance (bp)
BlCas9	N_4CN**AA**	*GAPDH*	CATCATATGAGGCAGCCTTCTC	GATTCTAA	
		TUA3	AGTTCCAGACCAACCTTGTGCC	CTACCCAA	
		SPL7	GCGGCACAAGGTGTGCGAGGTG	CACGCCAA	
		SPL17	CCGCCACAAGGTGTGCTACATG	CACGCCAA	
SpdCas9	N**GG**	*GAPDH*	GTATATAGCTTCCAACGCAA	GGG	-65
			GATTTGAATGGCAAGCTTAC	GGG	+50
		TUA3	ACCGCCTCCCTGAGGTTTGA	TGG	-45
			GCCTTCTCCGCAGAGATCAC	TGG	+29
		SPL7	GCACCCCTCCACCTGGCAAC	GGG	-77
			GCTTCTGCCAGCAATGCAGC	CGG	+55
		SPL17	GGTTGCGGCGTGGATCTGAG	CGG	-40
			GTTGGCAGAAGCGCTGCTCG	AGG	+24
enBlCas9	N_4CN**CA**	*TUA3*	GAGTTCCAGACCAACCTTGTGC	CCTACCCA	
	N_4CN**GA**		GTCTGGAACTCATTGACATCCA	CGTTCAGA	
	N_4CN**TA**		CAATGAGTTCCAGACCAACCTT	GTGCCCTA	
SpdCas9	N**GG**	*TUA3*	AGAACAGCACAAACAAGAAA	TGG	-132(N_4CN**CA**)<br>+91(N_4CN**GA**)<br>-128(N_4CN**TA**)
			GCCAAGTGCGACCCTCGCCA	CGG	+137 (N_4CN**CA**)<br>-178 (N_4CN**GA**)<br>+141 (N_4CN**TA**)
